# Effect of *Cymbopogon citratus* and Citral on Vascular Smooth Muscle of the Isolated Thoracic Rat Aorta

**DOI:** 10.1155/2012/539475

**Published:** 2012-05-22

**Authors:** R. Chitra Devi, S. M. Sim, R. Ismail

**Affiliations:** ^1^Department of Physiology, Faculty of Medicine, University of Malaya, Lembah Pantai, 50603 Kuala Lumpur, Malaysia; ^2^Department of Pharmacology, Faculty of Medicine, University of Malaya, 50603 Kuala Lumpur, Malaysia

## Abstract

*Cymbopogon citratus* has been shown to have antioxidant, antimicrobial, antispasmodic and chemo-protective properties. Citral, is the major constituent of *C. citratus*. This study investigated the effects of methanolic extracts of leaves (LE), stems (SE), and roots (RE) of *C. citratus* and citral on vascular smooth muscle and explored their possible mechanisms of action. The experiment was conducted using isolated tissue preparations, where citral, LE, SE, and RE were added separately into a tissue bath that contained aortic rings, which were pre-contracted with phenylephrine (PE). Citral, LE, and RE exhibited a dose-dependent relaxant effect on the PE-induced contractions. Citral appeared to partially act via NO as its vasorelaxant effect was attenuated by L-NAME. However, the effect of LE may involve prostacyclin as indomethacin reversed the relaxant effect of LE on the PE-induced contraction. Furthermore, citral, LE, and RE abolished the restoration of PE-induced contraction caused by the addition of increasing doses of calcium in both endothelium intact and denuded rings. These findings suggest that the relaxation effect of citral, LE, and RE is endothelium-independent and may be mainly by affecting the intracellular concentration of calcium. Citral may partially act through the NO pathway while a vasodilator prostaglandin may mediate the effect of LE.

## 1. Introduction

*Cymbopogon citratus*, commonly known as lemongrass, is a tropical perennial herb belonging to the family Poaceae (true grasses). It is commonly used in traditional Indian, Chinese, and Brazilian medicines [1].* Cymbopogon citratus* has been shown to be effective in the treatment of fever and infection, headaches, stomach aches, and rheumatic pain [2]. It is also reported to act as a sedative [2], antispasmodic [3], analgesic, anti-inflammatory [4], and antihypertensive [5] agents. However, the scientific evidence for its alleged therapeutic efficacy is still lacking. Furthermore, many of these reports concerning the effect of *C. citratus* described the function of only one particular part of the plant. The leaves decoction, for example, has been shown to have antioxidant property [6]. Meanwhile the stalk or stem of *C. citratus* was reported to have a small relaxation effect on perfused mesenteric arteries [7]. In this study we report the comparative effects of methanolic extracts of various parts of the plant on vascular smooth muscle. 

It is widely accepted that natural products of plant origin comprised a variety of components, and generally the major component is found to reflect well the biophysiological features of the extracts [8]. Phytochemical investigation of *C. citratus* showed that citral, 3-7-dimethyl-2,6-octadienal, is the major component of *C. citratus* [9, 10] and it is the most important member of the open-chain monoterpenoids. Studies have shown citral to be positive as anticlastogenic and anticancer agent [10, 11].

Most studies on natural products or drug receptor to signal transduction cascades, have used rats as the standard experimental animal model [12]. Similarly, studies on antihypertensive properties of plant extracts or natural products usually used rats, typically the spontaneously hypertensive rats (SHR) and their normotensive counterpart, Wistar-Kyoto (WKY) rats. The SHR strain is the most studied model of hypertension and cardiovascular diseases [13, 14]. As this strain was obtained by selective breeding of WKY rats, therefore, in all subsequent studies, WKY rats have been employed as controls for SHR [15]. Furthermore, studies involving vasodilatation or specifically vascular smooth muscles are often performed using isolated blood vessels. Isolated blood vessel pharmacology research allows scientists to explore mechanisms of action and to establish dose-response relationship for the analysis of the relative potency and efficacy of the drugs. Such studies also provide information in a controlled environment, without interference from changes in blood flow and sheer stress, extrinsic neural, or hormonal activity [16]. Isolated arterial ring preparations, commonly prepared from the rat aorta (thoracic region), are popular because aortic segments are relatively hardy and easily prepared [17].

Since we have previously reported a relaxant effect of citral and the extracts from *C. citratus* on visceral smooth muscle [3], the present study was aimed at investigating their effects on vascular smooth muscle and consequently provides further evidence for the antihypertensive effects of these test materials. The objective of the present study was therefore to examine the effects of citral and methanolic extracts of leaves, stems, and roots from *C. citratus* on the contraction of rat aortic rings. In addition, attempts were made to determine the mechanisms by which the test materials produce their respective vascular responses.

## 2. Methods

### 2.1. Ethical Approval

All the experimental procedures performed in this study were approved by the Animal Care and Use Committee of the Faculty of Medicine, University of Malaya (Reference number FIS/07/12/2006/RI(R)).

### 2.2. Drugs and Chemicals

For the extraction process, methanol was purchased from Merck, Germany. Meanwhile, for the *in vitro* aortic ring experiments the following chemicals were obtained from Sigma Chemical Co., USA: phenylephrine hydrochloride (PE), acetylcholine chloride (ACh), N*ω*-nitro-L-arginine methyl ester hydrochloride (L-NAME), indomethacin, and ethylenediaminetetraacetic acid disodium salt (EDTA). A stock solution of each drug except indomethacin, was prepared and diluted to the desired concentrations in distilled water. Dilution of indomethacin was prepared in 0.05% (w/v) Na_2_CO_3_. All drugs were prepared fresh on the day of the experiment. The concentrations of the drugs were expressed as the final molar concentration contained in the tissue bath. Krebs solution, pH 7.4, contains (mM) NaCl (118.0), KCl (4.7), KH_2_PO_4_ (1.2), CaCl_2_·2H_2_0 (2.6), NaHCO_3_ (25.0), MgSO_4_·7H_2_O (1.2), and glucose (11.1). The composition of Ca^2+^-free Krebs solution was similar to that of the normal Krebs solution but with EDTA 0.2 mM instead of CaCl_2_·2H_2_O.

Citral was purchased from Sigma Chemical Co., USA. A stock solution of citral was prepared in 2% (v/v) methanol in order to improve its miscibility in aqueous solution [3]. Test solution of citral was prepared fresh from the stock solution before each experiment, and the range of concentrations used in this study was from 0.00624 mM (0.0001%) to 6.24 mM (0.1%).

### 2.3. Plant Materials

Stalks of *C. citratus* were collected in the rural area of Beranang, Kajang, and the sample voucher (KLU 045309) was deposited in the Herbarium, Faculty of Science, University of Malaya.

#### 2.3.1. Plant Extraction

The collected *C. citratus* was cleaned and isolated into leaves, stems, and roots. They were separately oven-dried at 65°C and coarsely ground. The powdered materials of *C. citratus* (100 g) were extracted with 1000 mL of 70% methanol for 3 days with occasional shaking. A mixture of each part of the plant was filtered through Whatman qualitative grade 1 filter paper and this procedure was repeated 3 times. The extracts were then evaporated on a rotary evaporator (Gucci, Germany) at 40°C to eliminate the methanol. The extracts were then lyophilised and stored at −20°C until used. The yields were 12.34 g, 14.38 g, and 11.68 g, respectively, for the leaves (LE), stems (SE), and roots (RE). The powdered extracts were reconstituted with distilled water to the desired concentrations prior to use.

### 2.4. Animals

Male spontaneously hypertensive rats (SHRs) and Wistar Kyoto rats (WKY), weighing 250 to 300 g, were obtained from the University of Malaya Laboratory Animal Centre. The rats were kept in cages (5 per cage) and were comfortably maintained under controlled conditions. They were provided with normal rat chow (Gold Coin Feed Mills Sdn. Bhd., Malaysia) and water *ad libitum*. All rats were allowed to acclimatize in the animal holding room for a minimum period of two weeks before being used for any experiment.

#### 2.4.1. Preparation of Rat Aortic Rings

The rats were killed by cervical dislocation, and the thoracic aorta was isolated according to the procedure of Jain [18] with slight modifications. The thorax was opened to expose the aorta, and the descending thoracic aorta from the heart to diaphragm was dissected free. The isolated aortic tissue was immediately submerged in Krebs solution, aerated with a mixture of 95% O_2_ and 5% CO_2_ (carbogen). The surrounding connective tissue and fat were carefully trimmed off, and the aorta was then cut transversely into small rings of 2 to 4 mm length. Care was taken in isolating the tissue to avoid damage to the endothelium. However, in the endothelium-denuded group, the endothelium was removed by gently rubbing the luminal surface of the vessels back and forth several times with a polyethylene tubing (PE90). For each experiment a pair of rings from the same aorta (one with and the other without a functional endothelium) was used. Each of the rings was suspended, by means of two parallel stainless steel wires inserted into the lumen, in jacketed 10 mL tissue baths containing Krebs solution at 37°C and aerated with carbogen. The rings were each connected to an isometric transducer (Grass Instrument Co., Quincy, MA), and the transducer output was amplified and recorded continuously by a MacLab version 4.0 recording system (AD Instrument, Australia) coupled to a computer. The aortic rings were then progressively stretched to a basal tension of 1 g and allowed to equilibrate for 45 minutes. During this period the bathing solution was replaced every 15 minutes and, if needed, the basal tone was readjusted to 1 g tension before the start of experiment.

#### 2.4.2. Aortic Tissue Viability and Endothelium Intactness

At the end of equilibration period, each ring was challenged with 60 mM KCl in order to test for the contractility and viability of the rings. This procedure was repeated at least once or twice until the strength of two successive contractions differed by 10% or less. Once the viability of the rings was established, contraction was induced by adding PE (1 *μ*M) into the bath. When a plateau was obtained, ACh (1 *μ*M) was then added to test the endothelial integrity. The ability of ACh to induce at least 70% relaxation in the aortic rings of WKY rats and 50% for SHR is used to verify the intactness of the endothelium. A response of ≤10% ACh-induced relaxation was taken to indicate that the endothelium had been denuded [19].

### 2.5. Pharmacological Studies

#### 2.5.1. Effect of Citral and Extracts (LE, SE, and RE) on Vascular Tension of Endothelial Intact SHR and WKY Rat Aortic Rings on Phenylephrine- (PE-) Induced Contraction

In the first series of experiments, the aortic rings were prepared from both SHR and WKY rats. Following the primary equilibration, the relaxant effects of noncumulative addition of citral (0.00624 mM to 6.24 mM) on the submaximal PE-induced (1 *μ*M) aortic ring muscle tone were examined. A similar set of experiments was conducted using cumulative addition of extracts (1, 3, 10, 30, and 100 mg/mL) at 3-minute interval between successive additions. The control rings were similarly treated with PE but the corresponding vehicle (2% methanol for citral or distilled water for the extracts) was added instead. After each test, the rings were washed repeatedly at least three times with fresh Krebs solution and allowed to equilibrate for 30 minutes before testing with KCl for their viability. Only data from rings that remained viable at the end of the experiment were included for statistical analysis.

#### 2.5.2. Vasorelaxant Effect of Citral and Extracts on PE-Induced Contraction in Endothelial Intact SHR Aortic Ring Pretreated with L-NAME and Indomethacin

The contribution of endothelium-derived relaxing factors such as nitric oxide (NO) or cyclooxygenase-derived product such as prostacyclin (PGI_2_) in the vascular response elicited by test materials (citral and extracts) was examined by pretreatment of SHR aortic rings with either nitro-L-arginine methyl ester (L-NAME, 100 *μ*M) or indomethacin (10 *μ*M), respectively. The aortic rings were exposed to these modulating agents for 20 minutes prior to studying the vasorelaxant effects of the test materials on PE-induced contraction.

#### 2.5.3. Effect of Citral and Extracts on Extracellular Ca^2+^-Induced Contraction of Endothelial Intact and Denuded SHR Aortic Rings

In order to determine whether the inhibition of extracellular Ca^2+^ influx was involved in mediating the vasorelaxant effects of the extracts and citral, the experiment was also performed in Ca^2+^-free Krebs solution. After equilibration in the Ca^2+^-free Krebs solution for 20 minutes, a cumulative concentration-response curve for CaCl_2_ (0.1, 0.5, 1.0, 1.5, 2.0, and 2.5 mM) on PE-induced contraction was constructed. The construction of this concentration-response curve on the same aortic ring was repeated in the presence of test materials, which were given 20 minutes before PE was added. The data obtained in the absence of test materials served as control. The maximal tension produced by 2.5 mM calcium in the control group was considered as 100%, following which the concentration-response curves for the exogenously added calcium were constructed in the absence and presence of test materials.

### 2.6. Data Analysis

All results are expressed as the mean ± standard error of means (SEM) of 5 to 6 rats. The cumulative and noncumulative relaxant responses caused by the extracts and citral, respectively, were expressed as the percentage relaxation relative to the maximal contraction induced by 1 *μ*M PE. The differences in the response among the test substances were analyzed for statistical significance using Student's *t-*test for paired and unpaired observations and two-way analysis of variance. The PE-induced contraction in Ca^2+^-free Krebs medium was measured by expressing the magnitude of contraction obtained in the presence of test substances versus contraction obtained in the absence of test substances. In all cases the differences were considered significant if *P* < 0.05.

## 3. Results

### 3.1. Effect of Citral and Extracts (LE, SE, and RE) on Vascular Tension of Endothelial Intact Rat Aortic Rings on PE-Induced Contraction

**Figure 1:**
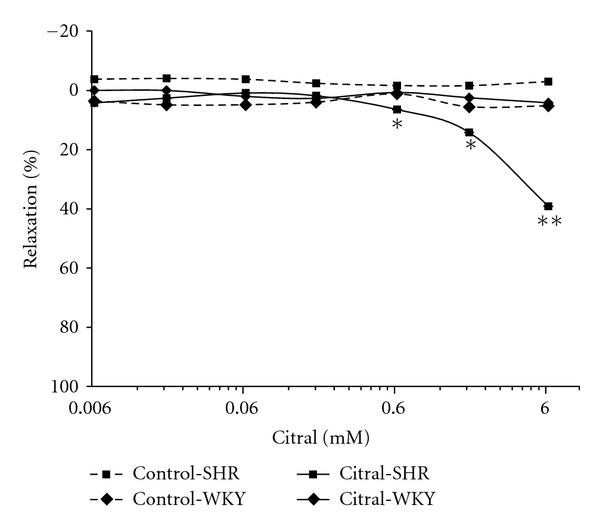
Dose-response curves for vasorelaxation effect of citral on PE-induced contraction in endothelial intact aortic rings of SHR and WKY rats. Values are shown as means ± S.E.M. with *n* = 6 rats, *P < 0.05, **P < 0.001 compared with the vehicle control, 2% methanol.

**Figure 2:**
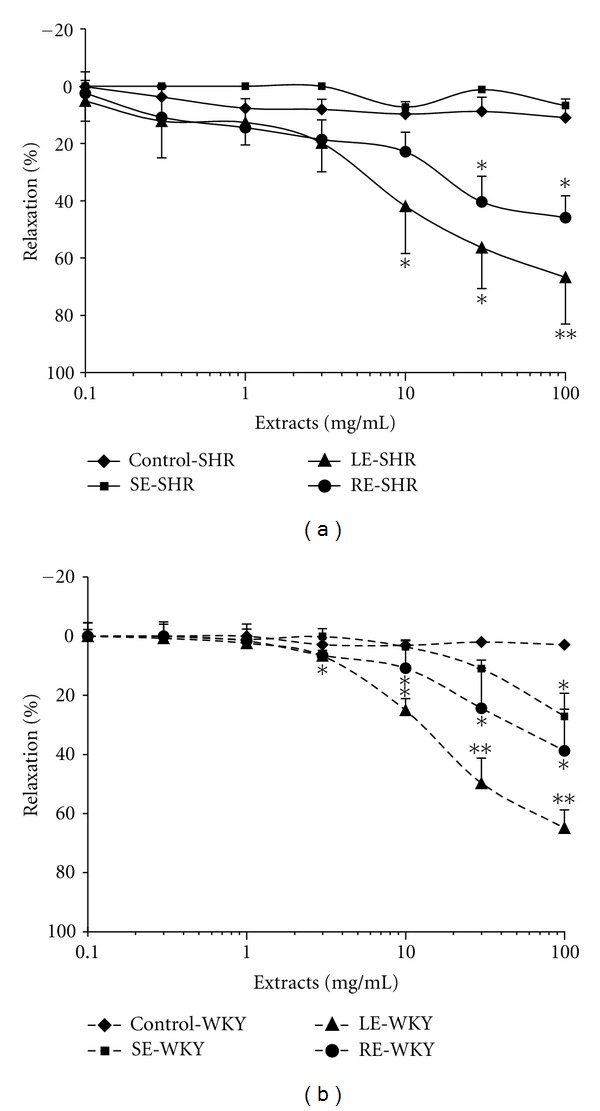
Dose-response curves for the vasorelaxation effect of extracts from leaves (LE), stems (SE), and roots (RE) on PE-induced contractions in aortic rings of (a) SHR and (b) WKY rats. Values are shown as means ± S.E.M. with *n* = 6 rats, *P < 0.05, **P < 0.001 compared with vehicle control, distilled water.

The isolated rat aortic rings were precontracted with PE to produce a sustained contractile response. As shown in Figure 1, citral at doses ranging from 0.00624 mM to 6.24 mM caused a concentration-dependent relaxation of PE-induced (1 *μ*M) contraction in SHR aortic rings compared to the vehicle control (2% methanol). The relaxation increased from 4.20 ± 0.02% to 39.13 ± 0.05%. However, citral at the same concentrations tested on WKY rat aortic rings did not cause a significant relaxation. In another series of experiments, cumulative addition of LE, and RE (from 0.1 mg/mL to 100 mg/mL) inhibited the contraction evoked by PE in a concentration-dependent manner in both SHR and WKY rat aortic rings (Figures 2(a) and 2(b)). The LE induced relaxation up to 66.76 ± 6.75% in SHR (IC_50 _= 22.8 mg/mL) and 64.92 ± 6.15% in WKY (IC_50 _= 29.5 mg/mL). Meanwhile, RE caused relaxation of up to 42.72 ± 5.79% in SHR and 38.8 ± 14.08% in WKY rat aortic rings. On the other hand, SE, showed weaker relaxation effects in both types of rats. The results clearly demonstrated that citral, LE, and RE produced relaxant effect on aortic ring smooth muscle. The question then arose as to how these test materials exert their relaxant effect. Moreover, the relaxant effect of citral and extracts at all concentrations used in this study was completely reversible suggesting that they are devoid of toxic effects. 

### 3.2. Pharmacological Investigation

#### 3.2.1. Vasorelaxant Effect of Citral and Extracts on PE-Induced Contraction in Endothelial Intact SHR Aortic Ring Pretreated with L-NAME and Indomethacin

**Figure 3:**
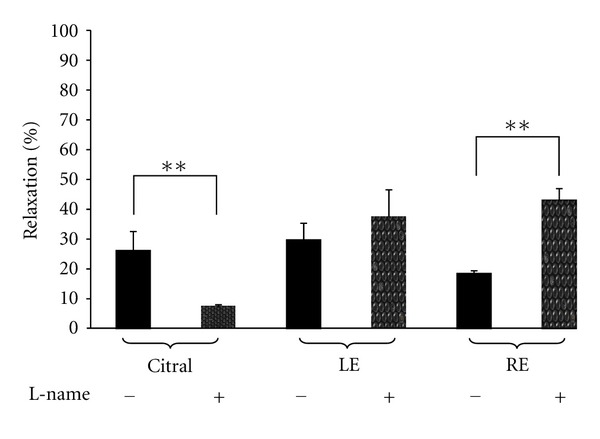
Effect of 6.24 mM citral and 30 mg/mL of extracts from leaves (LE) and roots (RE) of *C. citratus* on PE-induced contraction in the absence (−) and presence (+) of L-NAME (100 *μ*M) in endothelium intact aortic rings of SHR rats. Values are shown as means S.E.M. with *n* = 5-6 rats.

**Figure 4:**
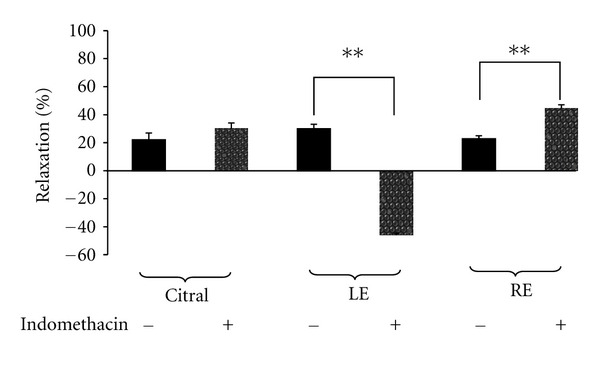
Effect of 6.24 mM citral and 30 mg/mL of extracts from leaves (LE) and roots (RE) of *C. citratus* on PE-induced contraction in the absence (−) and presence (+) of indomethacin (10 *μ*M) in endothelial intact aortic rings of SHR rats. Values are shown as means ± S.E.M. with *n* = 5 rats, **P < 0.001.

In the set of experiments conducted to verify the possible involvement of NO activation in the relaxant effect of citral, LE, or RE, the endothelium intact rings (E+) were Pretreated with L-NAME (100 *μ*M) followed by the addition of PE to induce contraction and then test materials. As shown in Figure 3, pretreating the rings with L-NAME caused the citral-induced relaxation to be significantly attenuated from 26.15 ± 6.38% to 7.19 ± 0.79% (*P* < 0.001). This finding indicated that citral elicited vascular relaxation via NO-dependent signalling mechanism, at least in part. In contrast, L-NAME significantly augmented the relaxant effect induced by RE from 21.26 ± 1.73% to 43.03 ± 3.92%. L-NAME also caused a similar change in the effect of LE but the increase did not reach the level of significance. In another set of experiments, pretreating the E+ aortic rings with indomethacin (10 *μ*M) produced variable effects: it did not alter the relaxant effect of citral but significantly enhanced the relaxant effect of RE, while it reversed the relaxant effect of LE to cause instead a significant increase in PE-induced contraction by 16.11 ± 1.78% (*P* < 0.001) (Figure 4).

#### 3.2.2. Effect of Citral and Extracts (LE and RE) on Extracellular Ca^2+^-Induced Contraction of Endothelial Intact and Denuded SHR Aortic Rings

**Figure 5:**
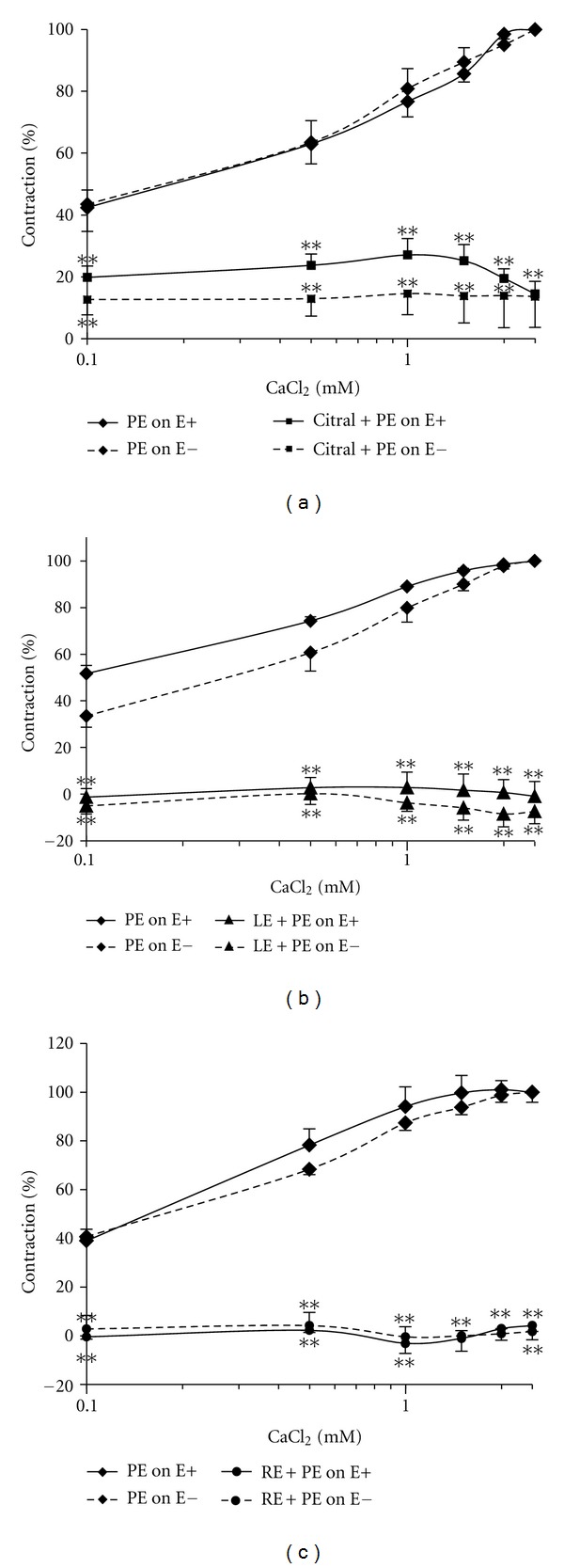
Dose-response curves for calcium in the presence and absence of (a) 6.24 mM citral and (b) 30 mg/mL of the extract from leaves (LE) and (c) roots (RE) of *C. citratus* on the PE-induced contraction in Ca^2+^-free Krebs solution in endothelial intact (E+) and endothelial denuded (E−) aortic rings of SHR rats. All the data were significant at **P < 0.001 compared to the PE-induced contraction in the corresponding E+ and E− aortic rings. Values were shown as means ± S.E.M. with *n* = 6 rats.

The addition of CaCl_2_ (0.1 mM to 2.5 mM) caused the transient PE-induced contraction of the endothelium intact (E+) or denuded (E−) aortic rings in Ca^2+^-free Krebs medium to increase in a dose-dependent manner (Figure 5). Preincubation of the aortic rings with citral, LE, and RE almost abolished this vasoactive effect of calcium. Thus, in the presence of citral, the addition of calcium (0.1 mM to 2.5 mM) caused minimal change to the contraction from 19.85 ± 3.66% at 0.1 mM to 14.47 ± 4.05% at 2.5 mM in E+ and from 12.62 ± 4.94% at 0.1 mM to 13.62 ± 10.00% at 2.5 mM in E− aortic rings. Similarly, LE and RE also greatly decreased (*P* < 0.001) the PE-induced contraction of aortic ring in Ca^2+^-free Krebs, and the addition of increasing doses of calcium did not augment the contraction.

## 4. Discussion

In the present study, the extracts (leaves, stems, and roots) from *C. citratus* were prepared as methanolic extracts. Despite the traditional practice of using aqueous extracts, methanol was chosen as the solvent so as not to miss any possible active compounds since it will dissolve both polar and some nonpolar constituents [20]. In addition, methanol is a common water-miscible solvent, and the solubility percentage in water is 100% with polarity index of 5.1 [21]. It needs to be pointed out that the methanol was evaporated and the dried extract residue was subsequently reconstituted in distilled H_2_O for all studies using the aortic ring preparation.

This paper reports the vasorelaxant properties of citral and methanolic extracts of leaf (LE), stem (SE), and root (RE) of *C. citratus* using isolated rat aortic rings and the possible mechanism(s) involved. The present observation of the vasorelaxant effect of citral on the PE-induced contraction in SHR aortic rings was in agreement with earlier studies that reported vasodilatory and antihypertensive effects of terpenoids [22]. A similar effect was shown by the leaf and root extracts suggesting that citral may be the main constituent in these extracts. The current study, however, indicates the presence of other compounds in both the leaf and root extracts since the vasorelaxant effect was observed in the aortic rings of both SHR and WKY rats, whereas citral showed the effect only in SHR.

Citral, LE, and RE significantly attenuated the contractile response induced by the selective *α*_1_-adrenoceptor agonist, PE, suggesting that these test materials may modulate the endothelial-derived relaxing factors (EDRFs) that include endothelial-derived NO (EDNO) and prostacyclin (PGI_2_)/prostaglandins [20, 23]. It is well established that NO is a major EDRF that plays a central role in the maintenance of vascular tone [24, 25]. When released from the endothelium, NO diffuses into the smooth muscle and then triggers the formation of cyclic guanosine monophosphate (cGMP), increasing the cGMP levels that lead to vasorelaxation. A reduced NO production by vascular endothelial cell is closely associated with endothelial disease or injury that has been proposed to be an important causative factor in cardiovascular diseases, especially in the development of arteriosclerosis and hypertension [26].

The involvement of NO in the relaxant effect produced by citral, LE, and RE in isolated aortic rings was investigated by incubating the E+ rings with the NO synthase inhibitor, L-NAME, prior to inducing contraction with PE and the subsequent addition of the test materials. The citral-induced smooth muscle relaxation on the PE-contracted rings was observed to be significantly reduced in the presence of L-NAME. These data provided evidence that NO may be involved in the vasorelaxant effect of citral. It also supports the hypothesis that citral may stimulate endothelial NO production and/or release. Whether citral is directly interacting with endothelial NO synthase or with other factors, which may increase the endothelial NO synthase activity, remains to be further investigated. Under the same condition, the relaxation caused by LE and RE was not attenuated. These results suggest that NO, a modulator of the vascular function [27], may partially play a role in the vasodilator effect of citral but not that of LE and RE. In fact, RE caused a significantly greater relaxant effect in the presence of L-NAME. The reason for this change remains to be elucidated.

Besides NO, prostaglandin (PG) is also produced by endothelial cells to counteract the vasoconstriction induced by the sympathetic nerve endings and humoral vasoconstrictors. After the discovery of EDRF, it was demonstrated that endothelial cells also can generate cyclooxygenase- (COX-) derived vasoconstrictor substances in canine veins and in arteries of SHR [28]. Thus, by secreting relaxing and constricting factors, the endothelium can induce dilation or constriction in response to sheer stress and a variety of endogenous vasoactive substances that are either produced systemically or generated locally by vascular tissues or circulating in the blood.

Prostacyclin (PGI_2_), a major vasodilatory COX product, is produced by the intimal layers of the vascular wall [27, 29]. In the present study, the involvement of PGI_2_ in the relaxant effect of the test materials on intact aortic ring preparations was investigated by preincubating the rings with indomethacin. Indomethacin is an inhibitor of COX and will inhibit the synthesis of various PGs including PGI_2_ and markedly inhibits the transient relaxation induced by arachidonic acid [30]. Indomethacin did not seem to affect the relaxant effect of citral. On the other hand, the relaxant effect produced by RE was significantly increased, thus, suggesting that PGI_2_ did not play an important role in the vasorelaxant response induced by RE. We cannot offer a good explanation for the increased vasorelaxant effect in this paper. Perhaps RE contains a non-PG vasodilator and a small amount of vasoconstricting PG such as thromboxane A_2_ such that in the presence of indomethacin the opposing vasoconstrictor response was removed giving rise to a net increase in vasorelaxant response.

Interestingly, the presence of indomethacin caused a reversal of the relaxant effect of LE, causing instead a contraction of the aortic rings. It could be postulated that the LE may have a vasoconstrictor as well as vasorelaxant agents as its constituents and that the relaxant effect is more dominant. The relaxant effect may partly be due to PGI_2_ or other PGs, and when the synthesis of PGs was inhibited by indomethacin, the non-PG vasoconstrictor effect became unmasked giving rise to contraction.

As with other muscles, the smooth muscle requires Ca^2+^ to contract. It is well established that the influx of external Ca^2+^ through specific Ca^2+^ channels or Ca^2+^ release from internal stores plays an important role in excitation-contraction coupling of smooth muscles. By binding to specific membrane receptors, PE induces Ca^2+^ influx through receptor-operated channels causing tonic contraction [31, 32] and stimulates the formation of inositol triphosphate (IP_3_) that binds to and opens specific IP_3_-receptor channels in the sarcoplasmic reticulum membrane, inducing Ca^2+^ release from intracellular storage sites and causing phasic contraction [32, 33].

When the aortic rings were incubated in a Ca^2+^-free Krebs solution and PE was added into the bath there was a transient increase in contraction due to release of Ca^2+^ from intracellular storage sites. The addition of increasing doses of CaCl_2_ solution into the organ bath caused a dose-dependent increase in contraction of both the E+ and E− aortic rings. However, when the rings were Pretreated with citral, LE, and RE, both the transient PE-induced contraction and the CaCl_2_-induced contraction were abrogated. Based on these findings, it may be postulated that citral, LE, and RE can either block the entry of Ca^2+^ from the extracellular space possibly via receptor-operated calcium channel or the Ca^2+^ release from intracellular storage sites. Further studies are required to understand the exact mechanism by which citral and the extracts affect the intracellular Ca^2+^ levels.

## 5. Conclusion

In summary, citral and the methanolic extracts of leaves and roots from *C. citratus* elicited relaxation on vascular smooth muscle. The present study demonstrated, for the first time, that citral is able to produce vasorelaxation in rat aorta, which appeared to be through NO pathway and blockade of calcium channels. Meanwhile, the methanolic extracts from leaves and roots were observed to exert vasorelaxation via blockade of calcium channels. The leaf extract may also cause relaxation of vascular smooth muscle through PGI_2_ since inhibition of its synthesis by indomethacin resulted in contraction. However, the exact mode of action on the vasorelaxant effect caused by these test materials remains to be investigated. The findings from this study provide a scientific basis for the use of this plant in traditional medicine and merits further investigations.
